# The Impact of Emotional Design on Multimedia Learning Outcomes: The Moderating Role of Task Difficulty

**DOI:** 10.3390/bs15030397

**Published:** 2025-03-20

**Authors:** Zhihong Liu, Zhenhong Wang

**Affiliations:** 1 School of Psychology, Shaanxi Normal University, Xi’an 710062, China; liuzhihong@snnu.edu.cn; 2 Shaanxi Provincial Key Research Center of Child Mental and Behavioral Health, Xi’an 710062, China

**Keywords:** multimedia learning, emotional design, cognitive load, task difficulty

## Abstract

Previous studies have demonstrated that emotional design can enhance multimedia learning outcomes. However, it is unknown whether task difficulty moderates the effect of emotional design on multimedia learning outcomes. This study aimed to explore this issue with the emotional design of multimedia learning materials in middle school mathematics probability knowledge learning. A 2 (emotional design vs. neutral design) × 3 (low, medium, and high task difficulty) between-subjects design was used to examine how emotional design affects multimedia learning outcomes in 180 middle school students under different levels of task difficulty. The results showed that the emotional design significantly elicited positive emotions among learners. Emotional design significantly only enhanced multimedia learning outcomes under medium-task-difficulty conditions (*p* < 0.001, *η_p_*^2^ = 0.09), but not under low- and high-task-difficulty conditions. These findings suggest that task difficulty is a crucial moderator of the effect of emotional design on multimedia learning outcomes.

## 1. Introduction

The emotional design of multimedia learning refers to the use of graphics with warm colors and face-like shapes in multimedia learning materials to facilitate learning performance by inducing a positive affective-motivation state in learners (Le et al., 2018; Um et al., 2012; X. Wang et al., 2023). The impact of emotional design on multimedia learning has been widely studied, but this research has yielded inconsistent results. Some studies have suggested that emotional design promotes learners’ positive affective-motivation states and improves multimedia learning outcomes (Chiu et al., 2020; Gong et al., 2017; Mayer & Estrella, 2014; X. Wang et al., 2023). Conversely, other studies have reported that emotional design does not significantly enhance multimedia learning outcomes (Heidig et al., 2015; Knörzer et al., 2016; Stárková et al., 2019). As researchers suggest, the effect of emotional design on multimedia learning outcomes may be moderated by several potential factors and thus may lead to inconsistent results (Brom et al., 2018; Wong & Adesope, 2021).

According to cognitive load theory (CLT), learners’ cognitive load, including the extraneous cognitive load (ECL) and intrinsic cognitive load (ICL), may impact multimedia learning outcomes (Kalyuga, 2011; Sweller, 2020). In particular, the ICL, which is determined by prior knowledge and task difficulty, may influence the degree to which learners’ limited cognitive resources are consumed and moderate the effect of emotional design on multimedia learning outcomes (Chiu et al., 2020; Kalyuga, 2011). Recent studies have confirmed that the ICL determined by learners’ prior knowledge significantly moderated the effect of emotional design on multimedia learning outcomes (Chiu et al., 2020; Shangguan et al., 2020). However, empirical research has not explored whether the ICL determined by task difficulty moderates the effect of emotional design on multimedia learning outcomes. The present study examined this issue, which is critical to understanding how emotional design can enhance multimedia learning outcomes and how the impact of emotional design varies with task difficulty.

### 1.1. Effects of Emotional Design on Multimedia Learning Outcomes

Multimedia learning refers to learning from a combination of pictures and words (Mayer, 2017). The Cognitive Theory of Multimedia Learning (CTML; Mayer, 2005) describes the process of how learners select, organize, and integrate information in the multimedia learning environment, with an emphasis on the role of cognitive processes. Expanding on the CTML, the Cognitive Affective Theory of Learning with Media (CATLM; Moreno & Mayer, 2007) emphasizes the influence of emotion and motivation on multimedia learning. According to the CATLM, the affective features of instructional messages can induce learners’ emotions, change learners’ motivation, and then influence learners’ cognitive processing and learning outcomes. As mentioned above, the emotional design of multimedia learning integrates emotional elements into multimedia learning materials, such as the use of warm colors and anthropomorphic illustrations (Um et al., 2012), to cultivate a positive affective-motivation state and facilitate cognitive engagement and then improve multimedia learning outcomes.

In the frame of the CATLM, empirical studies have investigated the impact of emotional design in multimedia learning. Initial studies demonstrated that warm-toned and anthropomorphic emotional designs in multimedia learning materials effectively elicited positive emotions, thereby enhancing both retention and transfer performance (Plass et al., 2014; Um et al., 2012). Subsequent research further established that emotional designs could independently improve either retention (Le et al., 2018; Mayer & Estrella, 2014; Uzun & Yıldırım, 2018) or transfer test scores (Bülbül & Kuzu, 2021; Gong et al., 2017; X. Wang et al., 2023). Additionally, X. Wang et al. (2023) found a positive correlation between higher levels of emotional design and improved learning outcomes. However, several studies have reported inconsistent results, highlighting that emotional design does not significantly facilitate either retention or transfer scores in multimedia learning (Heidig et al., 2015; Knörzer et al., 2016; Le et al., 2021; Stárková et al., 2019). For example, Heidig et al. (2015) employed emotional design using color schemes (classic vs. expressive esthetics) in website learning materials but found that this did not significantly impact learners’ emotional states or learning outcomes. Le et al. (2021) and Stárková et al. (2019), employing emotional design approaches and learning content that were nearly identical to Mayer and Estrella (2014), found that emotional design increased college students’ enjoyment or positive emotions, but did not significantly promote retention or transfer test scores.

Many factors may contribute to these discrepancies, including differences in the operationalization of independent and dependent variables and variations in research contexts. Specifically, moderator variables such as age, instruction time, language, culture, learning domain, and design approach can potentially influence the direction of emotional design’s effect on multimedia learning (Brom et al., 2018; Wong & Adesope, 2021).

### 1.2. Task Difficulty, Emotional Design, and Multimedia Learning Outcomes

As mentioned above, cognitive load comprises both ECL and ICL (Kalyuga, 2011). ECL refers to the load that is associated with cognitive processes that are extraneous to learning (Kalyuga, 2011). For example, poorly designed materials, distracting multimedia elements, or overuse of animations can consume a learner’s limited cognitive resources. Minimizing ECL proves advantageous for successful multimedia learning (Krieglstein et al., 2022). ICL is described as the load that is caused by the internal complexity of the learning materials (Kalyuga, 2011). Thus, this load is determined by the task difficulty and the learner’s domain-specific prior knowledge (Kalyuga, 2011). Low prior knowledge and high task difficulty lead to high ICL, and vice versa (Krieglstein et al., 2022). According to CLT, learners’ cognitive load, especially the ICL, may influence the degree to which learners’ limited cognitive resources are consumed and moderate the effect of emotional design on multimedia learning outcomes. Moreover, the Integrated Cognitive–Affective Model of Learning with Multimedia (ICALM; Plass & Kaplan, 2016) suggests that the interactive processes of emotion and cognition influence each other and may consume learners’ limited cognitive resources and also influence multimedia learning outcomes. Therefore, the ICL, which is determined by prior knowledge and a high task difficulty, may moderate the effect of emotional design on multimedia learning outcomes.

Learners’ prior knowledge, a key determinant of ICL, has been the focus of recent research exploring its moderating role in the relationship between emotional design and multimedia learning outcomes (Chiu et al., 2020; Knörzer et al., 2016; Shangguan et al., 2020). As researchers (Johnson et al., 2015) have suggested, students with high levels of prior knowledge have sufficient cognitive resources and relatively lower ICL, enabling them to efficiently select and process relevant information. For these learners, emotional design in learning may be unnecessary. In contrast, students with low levels of prior knowledge have insufficient cognitive resources and higher ICL in learning. These learners may benefit from the instructional support provided by emotional design, which helps to reduce cognitive load by guiding them to identify relevant information within visual representations in learning. Recently, Chiu et al. (2020) found that novice learners performed better with emotional design, whereas advanced learners performed better without emotional design. Therefore, prior knowledge levels may moderate the effectiveness of emotional design by influencing ICL.

Task difficulty is a crucial determinant of ICL, referring to the cognitive demands imposed by different tasks, and can directly influence the cognitive load that is experienced by learners at varying levels of task difficulty (Schneider et al., 2022). For tasks with high difficulty, the ICL will likely be elevated due to the insufficient development of relevant cognitive schemas and increased element interactivity (Sweller, 2020). Under this condition, learners may experience cognitive overload, which could hinder their ability to effectively process the learning material (Schneider et al., 2022). As a result, even though emotional design features may provide supportive cues, learners’ cognitive resources may be overwhelmed, potentially limiting the benefits derived from these emotional elements and leading to the disappearance of the effect of emotional design on multimedia learning outcomes. Conversely, for tasks with low difficulty, ICL is expected to be relatively low, as these tasks are less complex and require less cognitive effort. In such cases, emotional design is also unlikely to yield significant benefits, as the simplicity of the task may already result in high performance (J. Wang et al., 2020). For tasks of a medium difficulty, learners’ ICL will be at an optimal level, and they are likely to encounter an appropriate level of challenge, utilizing existing cognitive schemas to manage the cognitive load effectively (Krieglstein et al., 2022). This balanced cognitive load facilitates learners’ engagement with the material, enhancing their ability to process the content. Under this condition, emotional design may play a more significant role in improving learning outcomes. Emotional design could assist in directing learners’ attention to the most relevant information, reducing cognitive load, and thereby enhancing learning performance.

Taken together, different levels of task difficulty may moderate the effects of emotional design, but this has not yet been explored in the existing research. The present study examined whether and how the task difficulty determining the ICL in multimedia learning moderates the effect of emotional design on multimedia learning outcomes. Understanding task difficulty’s role is vital, as it influences the effectiveness of emotional design in multimedia learning environments.

### 1.3. The Present Study and Hypotheses

In summary, the present study aims to examine whether the effect of emotional design on multimedia learning outcomes is moderated by task difficulty, which is manipulated by the three levels (low, medium, and high) of junior statistics multimedia learning materials. This study posits the following hypotheses:

**H1.**  *Compared with a neutral design, the positive emotional design of multimedia learning will significantly induce and maintain positive emotions.*

**H2.**  *The emotional design of multimedia learning is expected to significantly improve learning outcomes under medium-task-difficulty conditions but not under low- or high-task-difficulty conditions.*

## 2. Materials and Methods

### 2.1. Participants and Design

This study employed a 2 × 3 between-subjects design featuring two factors: emotional design (emotional design vs. neutral design) and task difficulty (low, medium, high). A total of 180 students were recruited from a middle school in Northwestern China during the first semester of the 7th grade to control for prior knowledge, as the low, medium, and high task difficulty levels corresponded to the second semester of the 9th grade (see Section 2.2). Participants in this study had no prior knowledge of probability. They were randomly assigned to six groups, each consisting of 30 students: the neutral low-task-difficulty group (NL; 14 males; *M_age_* = 12.33; *SD* = 0.48); the emotional low-task-difficulty group (EL; 16 males; *M_age_* = 12.50; *SD* = 0.50); the neutral medium-task-difficulty group (NM; 14 males; *M_age_* = 12.50; *SD* = 0.48); the emotional medium-task-difficulty group (EM; 14 males; *M_age_* = 12.43; *SD* = 0.50); the neutral high-task-difficulty group (NH; 16 males; *M_age_* = 12.53; *SD* = 0.50); and the emotional high-task-difficulty group (EH; 17 males; *M_age_* = 12.63; *SD* = 0.49). Informed consent was obtained from all participants.

### 2.2. Design of Materials

This study utilized various middle school probability topics to manipulate the learning task difficulty. Three middle school mathematics teachers with over six years of teaching experience were invited to develop learning content and tests at three difficulty levels: low, medium, and high. Additionally, two senior teachers with over ten years of teaching experience in middle school mathematics were invited to review the difficulty levels of the learning content and tests. The developed learning content centered on probability topics from the middle school mathematics curriculum. The contents for low, medium, and high task difficulty corresponded to the topics of random events, probability calculations, and advanced probability calculations, respectively. According to the curriculum, these topics are covered in the second semester of the 9th grade.

To ensure comparability across difficulty levels, all learning materials followed a uniform structure and were delivered by the same teacher with over six years of experience. The teaching style, language, and instructional approach remained consistent across conditions. The materials were standardized in terms of question types, procedures, design, video format, duration, structure, and number of examples, with only the complexity of the probability problems varying. Test items aligned with the corresponding difficulty levels, and all followed the same response format and scoring criteria to ensure comparability in assessment.

An example of a low-difficulty topic is as follows:

“*A fair six-sided die has numbers 1 through 6 on its faces. What are the possible outcomes when the die is tossed?*”

(Answer: random event, 1, 2, 3, 4, 5, 6.)

An example of a medium-difficulty topic is as follows:

“*A bag contains 5 red balls and 3 green balls. One ball is randomly drawn, with a replacement. What is the probability of drawing one red ball and one green ball in two draws?*”

(Answer: the probability of drawing a red ball is 5/8, and drawing a green ball is 3/8.)

An example of a high-difficulty topic is as follows:

“*At an intersection, three cars can either go straight, turn left, or turn right. If these three possibilities are equally likely, what is the probability that at least two cars will turn left?*”

(Answer: the probability is 7/27.)

Following the learning content design, three versions of instructional videos, presented in grayscale with no additional design, were created for the NL, NM, and NH groups. A pre-experiment was conducted to confirm that the task difficulty of the materials met the required standards and to effectively differentiate between difficulty levels before the main study. Ninety students were recruited from a middle school in Northwestern China during the first semester of the 7th grade to control their prior knowledge and were randomly divided into NL, NM, and NH groups. Participants were asked to use the multimedia materials and complete the perceived task difficulty questionnaire and learning outcome tests (See Section 2.3).

The one-way ANOVA revealed significant effects of the group on the perceived task difficulty (*F* (2, 87) = 63.98; *p* < 0.001; *η_p_*^2^ = 0.60) and learning outcomes (*F* (2, 87) = 180.09; *p* < 0.001; *η_p_*^2^ = 0.80). Post hoc comparisons indicated that the perceived task difficulty in the low-difficulty group (*M* = 2.13; *SD* = 1.20) was significantly lower than in the medium-difficulty group (*M* = 4.43; *SD* = 1.43), with *t* (58) = −6.75, *p* < 0.001, and Cohen’s *d* = 1.74; meanwhile, the perceived task difficulty in the medium-difficulty group was significantly lower than that in the high-difficulty group (*M* = 6.20; *SD* = 1.54), with *t* (58) = − 4.61, *p* < 0.001, and Cohen’s *d* = 1.19. The learning outcomes were significantly higher in the low-difficulty group (*M* = 12.01; *SD* = 1.58) compared to the medium-difficulty group (*M* = 7.17; *SD* = 1.90), with *t* (58) = 10.73, *p* < 0.001, and Cohen’s *d* = 2.77, and they were significantly higher in the medium-difficulty group compared to the high-difficulty group (*M* = 3.57; *SD* = 1.69), with *t* (58) = 7.76, *p* < 0.001, and Cohen’s *d* = 2.00. These analyses were conducted specifically to assess whether the task difficulty levels were well differentiated and to ensure that the materials were appropriate for the subsequent experiment. The results suggested that the learning materials designed for the study can effectively manipulate the learning task difficulty and can be utilized in subsequent experiments.

**Figure 1:**
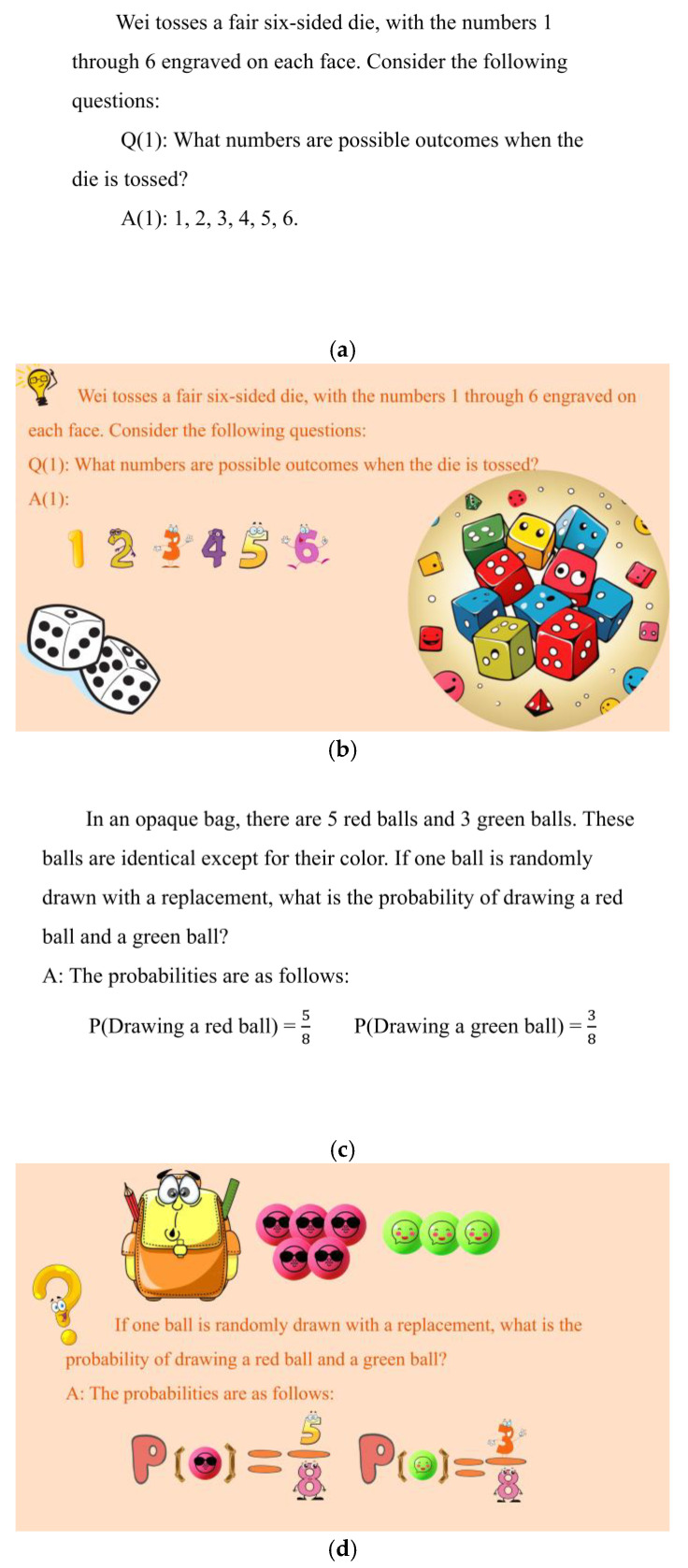

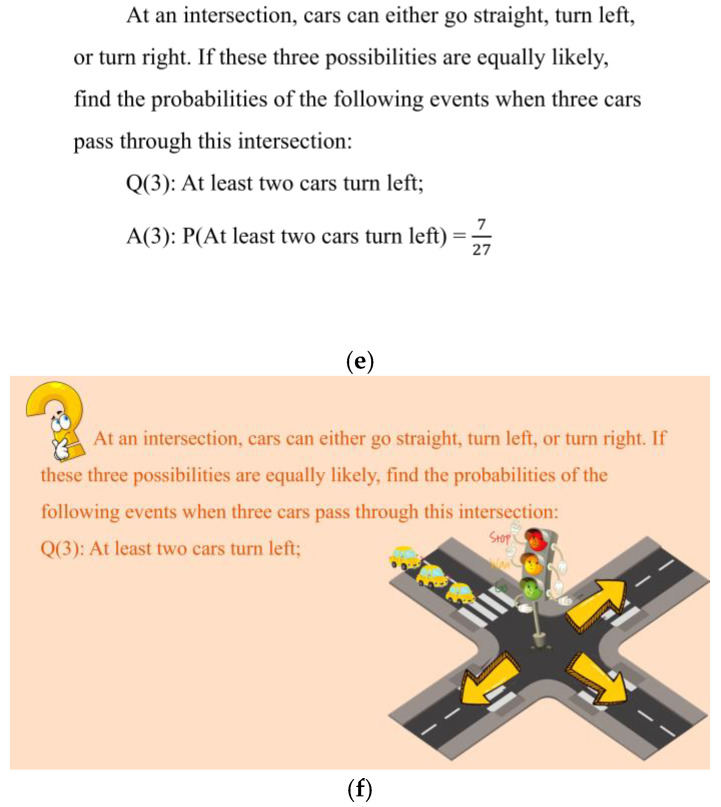
Screen capture of the multimedia videos showing different experimental conditions: (**a**) NL (neutral–low task difficulty), (**b**) EL (emotional–low task difficulty), (**c**) NM (neutral–medium task difficulty), (**d**) EM (emotional–medium task difficulty), (**e**) NH (neutral–high task difficulty), and (**f**) EH (emotional–high task difficulty). The instructional content was originally presented in Chinese during the actual experiment, with the English translation provided here for clarity.

Based on the NL, NM, and NH instructional videos, we created three different instructional video versions for the EL, EM, and EH groups. Informed by the findings of previous studies (Le et al., 2018; Mayer & Estrella, 2014; Um et al., 2012), the emotional design utilized in this study applied warm colors and anthropomorphic shapes to induce positive emotions without changing the learning content of the materials. The emotional design employed saturated, bright, warm color combinations, such as yellow, orange, and pink, for animation and visual design elements, along with round shapes instead of square ones for illustrations and characters. For instance, in the neutral design, the number 5 was presented as standard text, whereas in the emotional design, it was depicted in orange with anthropomorphic features. These manipulations were selected based on their empirically established emotional impact, ensuring that no additional learning content was introduced that could confound the results. The resulting six versions of the instructional videos were identical except for variations in the presence or absence of emotional design and task difficulty (see Figure 1).

### 2.3. Measures

Task difficulty was assessed using a 9-point perceived task difficulty questionnaire that asked “How difficult was the material to understand?”. This instrument is the most widely used method for evaluating learners’ perceived task difficulty (Kalyuga et al., 2000; Krieglstein et al., 2022). The questionnaire ensured consistency across participants in assessing the task difficulty and confirmed the effectiveness of the task difficulty manipulation in our experimental materials.

Positive emotions were assessed using the Positive Affect Scale (PAS), derived from the Positive and Negative Affect Schedule (PANAS; Watson et al., 1988). Ten positive emotions were measured using a 5-point Likert scale, ranging from “1” (indicating very slight or no presence) to “5” (indicating a high degree of presence). Cronbach’s α was calculated to be 0.93 in this experiment.

Learning outcomes were evaluated through tests that were aligned with the difficulty of the instructional material, comprising multiple-choice, fill-in-the-blank, and problem-solving questions, each with a total score of 15 points. Some examples are provided in the following: in low-difficulty tests, “Which of the following events are random, necessary, or impossible? 1. A shooter shoots once and hits 10 rings. 2. A cooked duck flies. 3. Three strips of wood with lengths of 3cm, 5cm and 6cm can form a triangle”; in medium-difficulty tests, “An opaque bag contains 4 red balls, 3 white balls, 2 yellow balls, these balls are the same except for different colors, touch 1 ball at random from the bag, the probability of touching a red ball is?”; and in high-difficulty tests, “On the table there are four cards with identical backs, the fronts of the cards are labeled with the numbers 4, −2, 1, and 3, turn the four cards face up and randomly select two of them, then the probability that the sum of the numbers on the two cards is a positive number is?”. Two independent raters evaluated the test scores. The inter-rater reliability on the low-, medium-, and high-task-difficulty tests was *r* = 1.00.

### 2.4. Procedure

The formal experiment was conducted in a laboratory setting through a computer program and a paper-and-pencil questionnaire. First, the researcher briefed the participants about the study and had them follow the instructions on the computer to learn about probability. Second, participants completed the demographic questionnaire. After that, depending on the grouping of the participants, the participants watched the instructional videos. After learning the material, participants were asked to complete a measure of perceived task difficulty and PAS. Finally, participants completed a learning test corresponding to their grouping. The entire process lasted approximately 50 min. At the end of the experiment, the participants were given a small gift.

## 3. Results

Table 1 shows the descriptive statistics for all variables.

### 3.1. Task Difficulty

We conducted a 2 (emotional design) × 3 (task difficulty) ANOVA to investigate whether emotional design and task difficulty had any effects on learners’ perceived task difficulty. The results showed that the main effect of task difficulty was significant, with *F* (2, 174) = 79.55, *p* < 0.001, and *η_p_*^2^ = 0.48; learners in the low-task-difficulty groups (*M* = 2.42; *SD* = 1.41) reported a significantly lower perceived task difficulty than those in the medium-task-difficulty groups (*M* = 4.53; *SD* = 2.04), with *t* (118) = −6.62, *p* < 0.001, and Cohen’s *d* = 1.20; meanwhile, learners in the medium-task-difficulty groups reported a significantly lower perceived task difficulty than those in the high-task-difficulty groups (*M* = 6.37; *SD* = 1.65), with *t* (118) = −5.42, *p* < 0.001, and Cohen’s *d* = 0.99. Neither the main effects of emotional design (*F* (1, 174) = 0.15; *p* = 0.70; *η_p_*^2^ = 0.001), nor the interaction effects were significant (*F* (2, 174) = 1.40; *p* = 0.25; *η_p_*^2^ = 0.016). The results indicated that the manipulation of task difficulty was successful.

### 3.2. Positive Emotions

We conducted a 2 (emotional design) × 3 (task difficulty) ANOVA to investigate whether the emotional design and task difficulty affected learners’ positive emotions. The results showed that the main effect of task difficulty was not significant, with *F* (2, 174) = 0.65, *p* = 0.53, and *η_p_*^2^ = 0.007. The main effect of emotional design was significant, with *F* (1, 174) = 59.20, *p* < 0.001, and *η_p_*^2^ = 0.25; learners in the emotional design groups (*M* = 3.59; *SD* = 0.80) reported significantly higher positive emotions than those in the neutral design groups (*M* = 2.75; *SD* = 0.65), with *t* (178) = 7.72, *p* < 0.001, and Cohen’s *d* = 1.15. The interaction effects were not significant: *F* (2, 174) = 0.88, *p* = 0.42, and *η_p_*^2^ = 0.01. The results indicated that the emotional design in this study was successful.

### 3.3. Learning Outcomes

**Figure 2:**
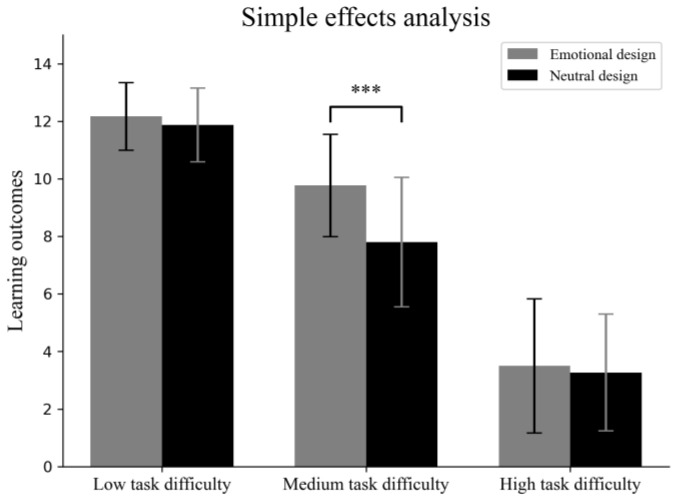
Results of interaction effect on learning outcomes (error bars represent standard deviation). *** *p* < 0.001.

We conducted a 2 (emotional design) × 3 (task difficulty) ANOVA to investigate whether the emotional design and task difficulty affected learners’ learning outcomes. The results showed that the main effect of task difficulty was significant, with *F* (2, 174) = 329.29, *p* < 0.001, and *η_p_*^2^ = 0.79. The main effect of emotional design was significant, with *F* (1, 174) = 9.02, *p* < 0.01, and *η_p_*^2^ = 0.05. The interaction effects were significant, with *F* (2, 174) = 4.17, *p* < 0.05, and *η_p_*^2^ = 0.05. A simple effects analysis (see Figure 2) showed that emotional design led to higher learning outcomes than neutral design under medium-task-difficulty conditions (*F* (1, 174) = 16.74; *p* < 0.001; *η_p_*^2^ = 0.09) but not under low-task-difficulty conditions (*F* (1, 174) = 0.39; *p* = 0.53; *η_p_*^2^ = 0.002) or high-task-difficulty conditions (*F* (1, 174) = 0.24; *p* = 0.63; *η_p_*^2^ = 0.001). The results indicated that task difficulty plays a crucial moderating role in the effect of emotional design on multimedia learning outcomes.

## 4. Discussion

This study builds on previous research that examined whether the effect of emotional design on multimedia learning outcomes is moderated by the task difficulty, which is divided into three levels (low, medium, and high) of junior statistics multimedia learning materials. This study validates our hypothesis that emotional design can effectively induce learners’ positive emotions (Hypothesis 1), but only under medium-task-difficulty conditions, to promote multimedia learning outcomes (Hypothesis 2).

Our study found that emotional design enhanced learning outcomes only under medium-task-difficulty conditions. Specifically, under medium-task-difficulty conditions, the emotional design induced positive emotions and significantly improved learning outcomes. The emotional design incorporating warm colors and anthropomorphic elements effectively induced positive emotions among learners, aligning with prior research (Le et al., 2021; Mayer & Estrella, 2014; Plass et al., 2014; Uzun & Yıldırım, 2018). Given that math tests evaluate learners’ ability to transfer knowledge, our findings align with a broad range of previous empirical studies (Gong et al., 2017; Plass et al., 2014; Um et al., 2012; X. Wang et al., 2023) and meta-analyses (Brom et al., 2018; Wong & Adesope, 2021). Under medium-task-difficulty conditions, our study observed significant effects of emotional design on enhancing learning outcomes. This can be attributed to the optimal relationship between learners’ cognitive load and the probability of learning success. According to CLT, a medium task difficulty presents an optimal challenge that engages learners without overwhelming their cognitive capacity (Kalyuga, 2011; Krieglstein et al., 2022). Although the mediation analysis results of this study indicate that positive emotions do not directly enhance learning outcomes (see Supplementary Material), this may be due to the presence of more complex underlying pathways. According to the CATLM and previous research (Moreno & Mayer, 2007; X. Wang et al., 2023), emotional design can elicit positive emotions in learners, which may, in turn, enhance learning motivation, increase cognitive engagement, or improve attentional focus on instructional content through indirect pathways. These intermediary processes may ultimately contribute to better learning outcomes, even if the direct effect of positive emotions on learning performance is not significant.

Importantly, this study found that emotional design only enhanced multimedia learning outcomes under medium-task-difficulty conditions and not under low- or high-difficulty conditions. This aligns with previous research (Javora et al., 2019; Knörzer et al., 2016; Le et al., 2021; Stárková et al., 2019). For example, Javora et al. (2019) found no benefits of emotional design for children, possibly due to a high task difficulty, while Le et al. (2021) and Stárková et al. (2019) observed no positive effects due to the low difficulty and simple content for college students. According to CLT, the task difficulty is a critical factor in determining ICL (Sweller, 2020). A high difficulty increases ICL through greater element interactivity and insufficient cognitive schema development (Kalyuga, 2011; Krieglstein et al., 2022), potentially leading to cognitive overload (Schneider et al., 2022). While emotional design may offer supportive cues, excessive cognitive demand can limit its benefits. Conversely, a low task difficulty involves less cognitive effort and lower ICL, allowing learners’ cognitive engagement to suffice without emotional design, reducing its impact (J. Wang et al., 2020). These findings contrast with the assumptions of the CATLM (Moreno & Mayer, 2007), which suggests that positive emotions boost cognitive engagement and learning outcomes. Our results show that cognitive engagement driven by positive emotions may not enhance learning under low- or high-task-difficulty conditions.

This study extends both the CATLM (Moreno & Mayer, 2007) and CLT (Sweller, 2020) by demonstrating that task difficulty, a crucial determinant of ICL, moderates the effectiveness of emotional design. Specifically, the effects of emotional design diminish or disappear under high- or low-task-difficulty conditions. This research contributes to these theories by incorporating task difficulty as a moderating factor, offering a more nuanced understanding of emotional design as being most effective when it enhances cognitive engagement without overwhelming cognitive resources. Moreover, this research provides practical guidance for educators and instructional designers, emphasizing the importance of aligning emotional design with task difficulty to optimize learning outcomes. In practice, educators can strategically implement emotional design elements, such as color and anthropomorphism, when designing learning materials for tasks that present an optimal challenge, ensuring that these elements support cognitive engagement rather than serve as distractions. This insight contributes new knowledge to the discourse on emotional design in multimedia learning, emphasizing the need to align emotional design strategies with task difficulty for optimal learning outcomes.

The present study has several limitations that should be addressed. First, in examining the moderating role of task difficulty on emotional design, the experimental design may be subject to potential floor and ceiling effects, which could obscure the true impact of emotional design on learning outcomes. Future research should employ more sensitive measurement tools, such as cognitive load measures derived from eye-tracking or EEG or refined experimental designs to better isolate these effects across varying task difficulty conditions. Second, while task difficulty was the primary manipulated variable, factors such as conceptual complexity and prior knowledge may have influenced the observed interaction effects. Although we controlled for instructional format and scoring, these factors may still introduce variability. Future studies should further isolate task difficulty by matching the content complexity, assessing prior knowledge, or incorporating cognitive load measurements. Third, while this study focused on task difficulty’s moderating role, future research should explore the specific mechanisms through which emotional and motivational factors mediate the relationship between emotional design and learning outcomes. Finally, given that this study focused on Chinese middle school students, future research should expand the sample to include learners from diverse geographical and cultural backgrounds to enhance the generalizability of the findings.

## 5. Conclusions

This study demonstrates that emotional design only enhances multimedia learning outcomes under medium-task-difficulty conditions, while it fails to enhance learning outcomes under low- and high-task-difficulty conditions. These findings emphasize the importance of task difficulty as a key moderator, offering a more nuanced understanding of how it influences the effectiveness of emotional design in multimedia learning.

## Figures and Tables

**Table 1:** Means and standard deviation of all variables for the six groups.

	NL	EL	NM	EM	NH	EH
Task difficulty	2.13 (1.38)	2.70 (1.39)	4.43 (2.01)	4.63 (2.09)	6.60 (1.79)	6.13 (1.48)
Positive emotions	2.67 (0.77)	3.58 (0.95)	2.94 (0.50)	3.57 (0.79)	2.64 (0.63)	3.61 (0.65)
Learning outcomes	11.87 (1.28)	12.17 (1.18)	7.80 (2.25)	9.77 (1.78)	3.27 (2.03)	3.50 (2.33)

## Data Availability

The data that support the findings of this study are available from the corresponding author upon reasonable request.

## Supplementary Materials

The following supporting information can be downloaded at: https://www.mdpi.com/article/10.3390/bs15030397/s1, Figure S1. Mediation analysis (**, *p* < 0.01); Table S1. Total effect, Direct effect, and Indirect effect. Reference (Horovitz & Mayer, 2021) is cited within the Supplementary Materials.
