# Supplementary material for: The Impact of Emotional Design on Multimedia Learning Outcomes: The Moderating Role of Task Difficulty
Source: Behav Sci (Basel). 2025 Mar 20;15(3):397. doi: 10.3390/bs15030397 (PMC11939454; doi:10.3390/bs15030397)
Supplement: Supplementary file 1 [file behavsci-15-00397-s001.zip › behavsci-3415613-supplementary.pdf]

Supplementary Materials

The results of our mediation analysis indicate that while emotional design significantly enhances both positive emotions and learning performance, the direct effect of positive emotions on learning outcomes is not statistically significant (See Figure S1 and Table S1). This suggests that the relationship between emotional design and learning outcomes may not follow a simple, direct mediation pathway through positive emotions alone. Instead, as highlighted in prior research (Horovitz & Mayer, 2021; Wang et al., 2023), emotional design may influence learning outcomes through more complex mechanisms, such as enhancing learning motivation, increasing cognitive engagement, or directing learners' attention more effectively to instructional content.

These findings align with the assumptions of CATLM, which posits that emotional design triggers affective-motivational processes that support deeper cognitive engagement, ultimately leading to better learning outcomes. However, the lack of a direct mediation effect suggests that alternative pathways, such as chained mediation involving motivation or attentional processes, may play a crucial role. This underscores the need for future research to explore these mechanisms further using refined experimental designs and advanced measurement techniques.

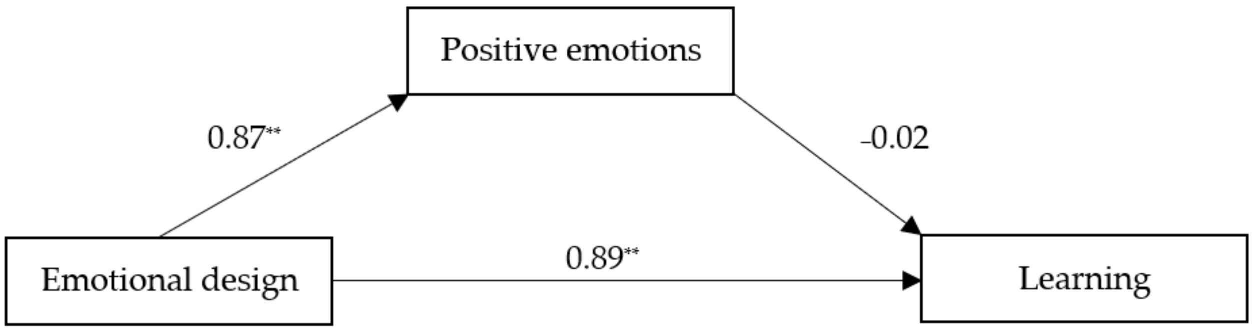

Figure S1. Mediation analysis (**, $p < 0.01$).

Table S1. Total effect, Direct effect, and Indirect effect.

	**Effect**	**se**	**LLCI**	**ULCI**
Total effect	0.87	0.23	0.01	0.41
Direct effect	0.89	0.26	0.01	0.37
Indirect effect	-0.02	0.10	-0.24	0.16

References:

Horovitz, T., & Mayer, R. E. (2021). Learning with human and virtual instructors who display happy or bored emotions in video lectures. *Computers in Human Behavior*, *119*, Article 106724. https://doi.org/10.1016/j.chb.2021.106724

Wang, X., Mayer, R. E., Han, M., & Zhang, L. (2023). Two emotional design features are more effective than one in multimedia learning. *Journal of Educational Computing Research*, *60*(8), 1991–2014. https://doi.org/10.1177/07356331221090845
